# Acute uveitis following zoledronic acid infusion

**DOI:** 10.4103/0974-620X.53046

**Published:** 2009

**Authors:** Krishna Kumar Rathnam, T. G. Sagar, Sanju Cyriac

**Affiliations:** Department of Medical Oncology, Cancer Institute (WIA), Chennai, India

Dear Sir,

Zoledronic acid is a bisphosphonate that has been widely used in oncological problems such as hypercalcemia of malignancy, multiple myeloma, metastatic carcinoma, and osteolytic bone metastases. Adverse reactions to zoledronic acid are usually mild and transient and similar to those reported for other bisphosphonates. Ocular side effects of bisphosphonates include conjunctivitis, anterior uveitis, episcleritis, and scleritis.[1,2] We report a patient who developed bilateral anterior uveitis within 24 hours, following treatment with zoledronic acid for metastatic breast cancer.

A 54-year-old woman with carcinoma of the left breast and metastatic bone disease came for her first monthly zoledronic acid infusion. Her past history was negative for any ocular disease, allergy, or connective tissue disease. She was also on oral etoposide and letrozole. The day following the injection of zoledronic acid (4 mg in 100 ml normal saline over 15 minutes), she developed bilateral conjunctival suffusion and edema of both eye lids. There was circumciliary congestion and pain during movement of both eye balls. Anterior segment flare was present, cells were 2+, fresh keratic precipitates were observed in the lower half of cornea, but no vitreous inflammation was observed. Ptosis and proptosis were absent. Vision, intraocular pressure, and rest of the examination was normal. A diagnosis of acute anterior uveitis secondary to zoledronic acid was made and she was treated with topical prednisone and atropine after which there was prompt resolution of all symptoms. She was continued on the same for five weeks on a tapering basis. She received no further doses of zoledronic acid.

Zoledronic acid is the most widely used bisphosphonate for metastatic bone disease and osteoporosis because of its relative higher potency and short infusion time. It binds to the hydroxyapatite and accumulates in the bone, thus inhibiting osteoclast migration and maturation. Side effects of zoledronic acid are usually mild which include a syndrome similar to flu; consisting of fever, chills, bone pain, and arthralgias. Ocular complications such as severe acute anterior uveitis, episcleritis, scleritis, and orbital inflammation requiring topical and at times even systemic steroid therapy[1,2] have been reported as a complication of pamidronate (another biphosphonate), but only rarely with zoledronic acid.[3] In patients with Paget′s disease of bone, pamidronate treatment is given for longer periods at higher doses that may heighten sensitivity to the drug.[2]

It has been suggested that the secretion of bisphosphanates into tears may cause conjunctivitis; however, bisphosphonates also trigger the release of cytokines, interleukin 1, and interleukin 6, along with other acute-phase proteins mediating ocular inflammation.[4] Ryan *et al*. proposed that these cytokines can cause extraocular muscle inflammation, resulting in orbital inflammatory disease.[5] The close temporal relationship between zoledronic acid infusion and the onset of ocular symptoms in our patient is in agreement with bisphosphonate-related ocular inflammation. Rapid response to drug withdrawal and topical steroid therapy also supports a drug-related etiology for this ocular process. Patients receiving zoledronic infusion therapy should be instructed to immediately report any eye complaints to their physicians. Prompt initiation of topical steroids is essential, as presumptive therapy with antibiotics only and delays in starting steroids can cause significant visual deterioration.[6]
